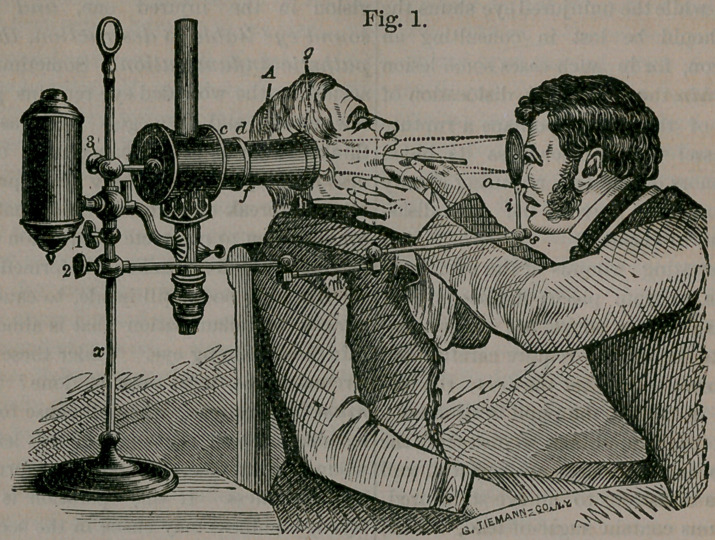# The Laryngoscope

**Published:** 1875-07

**Authors:** 


### THE LARYNGOSCOPE.

We have from time to time given illustrations of the ingenious apparatus that is employed in searching for and determining disease in various parts of the body, in order that the non-professional reader may learn how well every skillful surgeon is equiped, in these progressive times, to discover diseased action in the hidden cavities of the body.

We have, in a former number, described the ophthalmoscope, the wonderful instrument that enables us to examine the interior of the eye and to recognize diseases of the optic nerve or of the internal membranes or humors of the eye, and we now come to explain an equally useful and ingenious implement, named the *laryngoscope*, used for examining the *larynx*, or speaking apparatus, as well as the throat, wind-pipe, and posterior naries.

This instrument is especially useful to the surgeon in detecting causes for hoarseness and loss of voice, enabling him to illuminate the *larynx* and receive a reflection of all its parts upon the neat little mirror used for the purpose. In this manner paralysis, enlargement, or growths upon the vocal cords can be detected, and suitable treatment directed to the parts.

Fig. 1.

The above instrument is the one most used, and is considered the most perfect of the numerous forms of laryngoscopes now in use. It is named Tobold's Illuminator, after the celebrated surgeon, its inventor. It consists of a German student's lamp, enclosing the chimney of which, is a brass tube, A, supported by the stem of the lamp at 3. Inside the tube, at *d*, *c*, are two bi-convex lenses, and still a larger one, at the end of the tube, at *g*.—These lenses condense the light upon the concave mirror, *o*, which reflects it into the mouth, where it is again reflected from a small, plain mirror, attached to a handle, as seen held in the surgeon's hand. This small mirror is so held, by the surgeon making the observation, at the back of the throat, as to throw the light upon the larynx and to receive an image of it at the same time, which is seen through the small hole in the concave reflector through which he is looking.

In this manner, all parts of the throat, speaking-apparatus, wind-pipe, and the mouths of the eustachian tubes, leading from the middle ear, can be inspected, only varying the small hand-mirror to suit the point to be explored.

The instrument can also be used in examining the ear, and the one in our own possession is employed as an ophthalmoscope for examining the internal eye, preferring it to the ophthalmoscopes ordinarily used for this purpose; so it is at once seen that it becomes indispensible to every well equiped surgeon's outfit.

Previous to the introduction of this instrument, it was impossible to treat successfully the various ailments of the speaking apparatus; and the subjects of such difficulties were forced to speak in a whisper, or with a creaky, broken voice, for the reason that no means were at hand by which the cause could be detected. But now we have in our possession the means for discovering the smallest excrescences that are often found attached to the vocal cords, and so interfering with the clearness of the voice, and enabling us to remove them by suitable instruments under the guide of the Laryngoscope. Under the same direction, we are permitted to treat the various ailments of the throat, which too often result in more serious diseases, and is as well, a fruitful cause for certain forms of deafness. In short, the benefits that have resulted to mankind from the skillful use of the laryngoscope are not easily estimated, and should make us thankful for the men whose scientific attainments and inventive genius have placed it at our disposal.

DOCTOR'S CARRIAGES.—A venerable physician of Philadelphia, Dr. Condie, the author of a book on Diseases of Children, would never keep a carriage, nothwithstanding his large practice, and is made responsible for this *mot:* "If a doctor drive one horse, it indicates physical weakness; if he drive two, mental weakness."

We wonder what the old doctor would have to remark could he wake up and witness the elegant equipages, driven by the liveried servants of Prof. Gross, and other distinguished physicians of Philadelphia? The handsomest turn-outs in the Quaker city are owned by the doctors.

## Figures and Tables

**Fig. 1. f1:**